# A 3D Cell Culture Model Identifies Wnt/*β*‐Catenin Mediated Inhibition of p53 as a Critical Step during Human Hepatocyte Regeneration

**DOI:** 10.1002/advs.202000248

**Published:** 2020-06-23

**Authors:** Nuria Oliva‐Vilarnau, Sabine U. Vorrink, Magnus Ingelman‐Sundberg, Volker M. Lauschke

**Affiliations:** ^1^ Department of Physiology and Pharmacology Karolinska Institutet Stockholm 171 77 Sweden

**Keywords:** alternative splicing analysis, dedifferentiation, liver regeneration, proteomics, transcription factor motif analysis, transcriptomics

## Abstract

The liver is a highly regenerative organ. While mature hepatocytes under homeostatic conditions are largely quiescent, upon injury, they rapidly enter the cell cycle to recover the damaged tissue. In rodents, a variety of injury models have provided important insights into the molecular underpinnings that govern the proliferative activation of quiescent hepatocytes. However, little is known about the molecular mechanisms of human hepatocyte regeneration and experimental methods to expand primary human hepatocytes (PHH). Here, a 3D spheroid model of PHH is established to study hepatocyte regeneration and integrative time‐lapse multi‐omics analyses show that upon isolation from the native liver PHH acquire a regenerative phenotype, as seen in vivo upon partial hepatectomy. However, proliferation is limited. By analyzing global promoter motif activities, it is predicted that activation of Wnt/*β*‐catenin and inhibition of p53 signaling are critical factors required for human hepatocyte proliferation. Functional validations reveal that activation of Wnt signaling through external cues alone is sufficient to inhibit p53 and its proliferative senescence‐inducing target PAI1 (SERPINE1) and drive proliferation of >50% of all PHH. A scalable 3D culture model is established to study the molecular and cellular biology of human hepatocyte regeneration. By using this model, an essential role of Wnt/*β*‐catenin signaling during human hepatocyte regeneration is identified.

## Graphical abstract

This study reports an ex vivo model of human liver regeneration using a 3D organotypic culture system. Using in‐depth time‐lapse transcriptomic, proteomic, and transcription factor activity analyses, it is identified that Wnt/*β*‐catenin signaling acts as a central gatekeeper of human hepatocyte proliferation by modulating activity of the p53‐SERPINE1 axis.

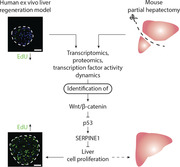

## 1 Introduction

The liver is unique among the visceral organs in its astounding capacity to regenerate after injury. Most information about liver regeneration stems from the 2/3 partial hepatectomy (PHx) model in rodents, in which the left lateral, left medial, and right medial hepatic lobes are surgically removed. In this paradigm, the murine liver regenerates to its original size within 7–10 days through a combination of hepatocyte hypertrophy and hyperplasia.^[^ ^1^, ^2^ ^]^ Elegant lineage tracing studies and genetic manipulations of PHx mice have unveiled important signaling pathways involved in liver regeneration, including growth factor, Hippo‐ and Wnt signaling.^[^ ^3^ ^]^

For end‐stage liver disease, orthotopic liver transplantation (OLT) and, more recently, repopulation with transplanted hepatocytes^[^ ^4^ ^]^ constitute the current treatment options. However, both methods are limited by a paucity of transplantable material. Furthermore, while outcomes have improved significantly with 10‐year survival around 70%, allograft recipients require the life‐long use of immunosuppressants and still lose an estimated 7 life years.^[^ ^5^ ^]^ Exploiting the regenerative potential of liver cells provides an appealing strategy to overcome these challenges on multiple fronts. First, the induction of *ex vivo* expansion of donor hepatocytes can ameliorate the shortage of available liver cells. Second, parsing the molecular mechanisms and dynamics of human liver regeneration might provide important cues for the optimization of stem cell differentiation protocols, which could eventually open new avenues for the transplantation of autologous hepatic material. Importantly however, our current molecular understanding of liver regeneration is primarily derived from rodent models. Recently human organoid cultures have been reported in which liver cells are capable of transdifferentiation and nearly unlimited proliferation.^[^ ^6^, ^7^, ^8^ ^]^ However, these models require scaffolding and complex treatment regimens with small molecule and growth factor cocktails, which result in selection and proliferation of only a small minority of liver cells.

It is well‐established that primary human hepatocytes (PHH) rapidly lose their phenotype in conventional monolayer culture on collagen‐coated plastic within few hours, a process termed dedifferentiation. Mechanistically, it is thought that plating PHH on stiff substrates with high Young moduli activates mechanotransduction through the focal adhesion kinase (FAK)‐Rho‐ROCK signaling axis^[^ ^9^, ^10^ ^]^ and triggers major changes of the non‐coding transcriptome,^[^ ^11^ ^]^ followed by loss of expression of hepatic marker genes. In stark contrast, once established, the molecular phenotypes of PHH in organotypic 3D culture models, such as spheroids, bioreactors, or liver‐on‐a‐chip systems, are stable for multiple weeks.^[^ ^12^, ^13^ ^]^ However, the molecular cues and signaling networks necessary for the maintenance of mature hepatic gene expression profiles have not yet been identified.

Here, we investigate the underlying mechanisms, using a systems biology approach based on integrative time‐lapse multi‐omics profiling of 3D spheroid cultures at the transcriptomic, proteomic and transcription factor (TF) activity level. Surprisingly, we found that during spheroid formation, PHH undergo dedifferentiation to a similar extent as in 2D monolayers affecting thousands of genes. Dedifferentiation in 3D culture however was only transient and, importantly, closely recapitulated the hepatocyte regenerative response upon PHx in mice on transcriptomic and proteomic levels. PHH during spheroid aggregation enter the cell cycle, alter their metabolic configuration and activate important signaling cascades, such as Hippo and growth factor signaling, corroborating its recapitulation of the liver regenerative program. By integrating these comprehensive omics data sets with global analyses of regulatory motif activities, we identify p53 as a crucial gatekeeper of the human liver regenerative program and show that activation of Wnt/*β*‐catenin signaling in 3D culture is sufficient to inhibit p53 and drive proliferation in >50% of adult PHH.

## 2 Experimental Section

### 2.1 Cell Culture

Cryopreserved PHH from five different donors (**Table** **1**) were commercially acquired from BioIVT (Maryland, US). The supplier sought informed consent from each prospective donor or the subject's legally authorized representative and these forms, along with their corresponding protocols, were reviewed and approved by the appropriate regulatory and ethics authorities in accordance with HHS regulations for the protection of human subjects (45 CFR §46.116 and §46.117) and Good Clinical Practice (ICH E6). Cells were thawed and seeded in PHH culture medium as previously described.^[^ ^14^ ^]^ For 2D PHH culture, cells were seeded at 3,5×10^5^ cells per mL onto 12 and 24‐well plates (Corning) coated with rat tail collagen I (Corning) in PHH culture medium. PHH were allowed to attach for 2 h, after which the medium was replaced with serum‐free PHH culture medium. Cells were maintained for 7 days with medium change every 48–72 h. For Wnt signaling activation, PHH spheroids were seeded in PHH culture medium containing 3 µm CHIR99021 (Tocris). For EdU DNA synthesis labeling, EdU was incorporated into the culture medium at 10 µm from seeding and EdU incorporation rates were assessed after 7 days.

**Table 1:** Demographic and medical information of the used primary human hepatocytes donors

Donor	Sex	Race	Age	Cause of death	Relevant medical and social history
1	Male	Caucasian	22	Intracerebral hemorrhage	Smoker, hypertension, 2 kidney transplants 12 and 2 years pre mortem
2	Female	African	27	Anoxia	Respiratory disease, Ventilator dependent quadriplegia
3	Female	Hispanic	30	Head trauma	Smoker, opiate dependency, 1–2 beers per day
4	Female	Caucasian	39	Anoxia	Smoker, hypertension, narcotic dependency
5	Male	Hispanic	25	Head trauma	Smoker, 6+ beers per day

### 2.2 RT‐qPCR Analyses

Total RNA was isolated from 24 pooled spheroids. Per sample 400 ng of RNA were reverse‐transcribed using SuperScript III reverse transcriptase (Invitrogen). qRT‐PCR reactions were carried out using the Taqman Universal PCR mix (Thermo Fisher) and appropriate TaqMan probes (Table S1, Supporting Information) on a 7500 Fast Real‐Time PCR system (Applied Biosystems). Expression levels were analyzed using the ΔΔ*Ct* method.

### 2.3 RNA‐Sequencing and Data Analysis

Total RNA was isolated from 96 spheroids (3D) or one 12‐well plate (2D culture). Bulk RNA sequencing (poly‐A) of a minimum of 100 ng total RNA was performed by the National Genomics Infrastructure (NGI) facility at Science for Life Laboratory, Stockholm, Sweden. Analyses were performed in *n* ≥ 3 independent experiments as indicated using cells from four different donors. Genes with an average number of fragments per kilo base per million mapped reads (FPKM) >1 across all samples were analyzed using Qlucore (Lund, Sweden). Multiple testing correction was performed using the Benjamini–Hochberg method with false discovery rates (FDRs) as indicated. KEGG pathway analyses were carried out using WebGestalt.^[^ ^15^ ^]^

### 2.4 Alternative Splicing Analysis and Transcription Factor Activity Analyses

Alternative splicing isoform abundance changes were analyzed using rMATS.3.2.5.^[^ ^16^ ^]^ The output was analyzed using the maser package from Bioconductor. Isoforms were considered as significantly abundant when FDR <0.05 and ΔPSI >10%. Transcription factor (TFs) activity profiles over spheroid culture and mouse liver regeneration timecourse datasets were obtained using the ISMARA algorithm.^[^ ^17^ ^]^ TFs were considered transiently upregulated when activity peaked within the first 3 days, and transiently downregulated when the minimum was reached in the same timeframe.

### 2.5 Tandem Mass Tag (tmt)‐Based Proteomics and Data Analysis

Total protein was isolated from 192 spheroids and lysates were analyzed by mass spectrometry analysis at the Clinical Proteomics Mass Spectrometry facility (Science for Life Laboratory, Stockholm, Sweden). Proteins with a Protein Spectrum Match (PSM) level of >1 in all analyzed samples were used for differential expression analyses, which were performed using Qlucore (Lund, Sweden).

### 2.6 Immunohistochemistry and Imaging

Spheroids were fixed in 4% paraformaldehyde at room temperature for 2 h and subsequently preserved in 30% sucrose overnight at 4 °C. Spheroids were then embedded in Tissue‐Tek OCT (Sakura) and cryosectioned into 10 µm thick sections. Sections were blocked and permeabilized for 2 h at room temperature in 5% BSA and 0.25% X‐Triton (Sigma). Sections were incubated overnight at 4 °C with primary antibody and subsequently for 2 h with secondary antibody at room temperature (Table S2, Supporting Information). Mounting was done using ProLong Gold Antifade Mountand with DAPI (Life technologies) and the stained sections were imaged on a Zeiss LSM800 Confocal.

### 2.7 Western Blot

A minimum of 96 spheroids were collected for protein isolation and resuspended in RIPA buffer (Thermo Fisher) supplemented with protease inhibitor and phosphatase inhibitors (Merck). Protein determination was carried out using DC protein assay (Bio‐Rad) and a total of 25 µg of protein were loaded for SDS‐PAGE gel electrophoresis. Membranes were blocked in 5% skim milk solution. Primary and secondary antibody incubations in 3% skim milk solution were performed overnight at 4 °C and 2 h at room temperature, respectively. Supersignal West Femto Maximum Sensitivity Substrate (Thermo Scientific) was used for band detection in a Chemidoc (Bio‐Rad) instrument.

### 2.8 Statistics

For human RNAseq data unsupervised hierarchical clustering was performed in Qlucore Omics Explorer 3.2 (Qlucore, Lund, Sweden). Differential gene expression analysis across time points was carried out using F‐tests (ANOVA), while comparisons between two groups were conducted using heteroscedastic two‐tailed *t*‐tests. Multiple testing correction was performed using the Benjamini–Hochberg method with significance at a false discovery rate of 0.01 unless otherwise specified. For human proteomics data, proteins were considered to be differentially expressed when *p* < 0.05 (heteroscedastic two‐tailed *t*‐test) and fold‐change >2 compared to controls. For spheroid volume calculations, spheroid diameters were determined from brightfield microscopy images using ImageJ and spheroid volume was calculated as sphere volume (*V* = 3/4(*π*)r^[^ ^3^ ^]^). A minimum of *n* = 10 spheroids were evaluated per experiments and average values for *n* = 6 experiments were calculated. For EdU incorporation measurements, a minimum of *n* = 8 spheroid sections per experiment were evaluated and average values for *n* = 5 experiments were calculated. No outliers were eliminated and all obtained data was included into the analyses.

## 3 Results

### 3.1 Loss of Cell–Cell Contacts Rather than Plating Drives Dedifferentiation of Primary Human Hepatocytes

In monolayer culture PHH rapidly dedifferentiate upon plating onto rigid substrata, whereas organotypic 3D cultures are considered as phenotypically stable. Little is known about the mechanisms and pathways underlying the phenotypic stability in 3D culture, albeit studies in cell lines implicated cell–cell contacts in the maintenance of hepatic differentiation.^[^ ^18^ ^]^ To investigate the molecular consequences of cell–cell interactions, we profiled the dynamics of transcriptomic signatures of mature hepatocytes during the formation of organotypic 3D cultures.

**Figure 1:**
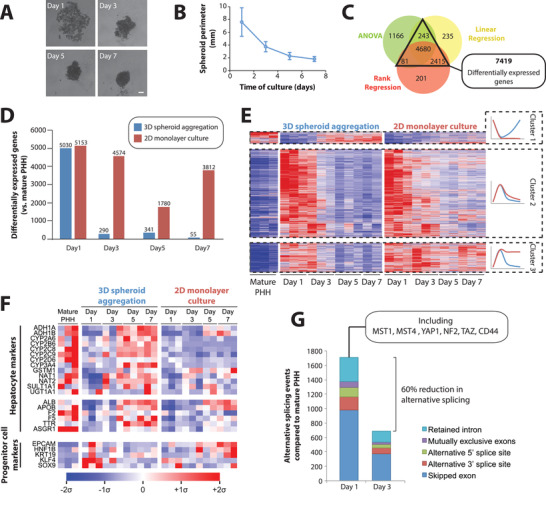
The phenotype of primary human hepatocytes in 3D spheroid culture is highly plastic. A) Time series of brightfield images of primary human hepatocytes (PHH) during the aggregation process. Scale bar = 100 µm. B) PHH cell aggregates compact substantially over the course of 7 days in spheroid culture. *N* = 29–55 spheroids per time point. Error bars indicate S.D. C) Time series RNA‐Seq analysis of PHH cultured as 3D spheroids or 2D monolayers, identified 7419 differentially expressed genes (DEGs) compared to mature PHH by at least two established statistical methods (*q* < 0.01; see Experimental Section for details). D) DEGs for 2D and 3D cultures at each evaluated time point compared to mature PHH (*t*‐test, *q* < 0.05). *N* = 3 with the exception of 5 days 2D and 7 days 3D culture for which only two samples were available for technical reasons, likely resulting in a lower number of DEGs for these conditions. E) Heatmap visualization of DEGs. Note the formation of three clusters. Cluster 1 is only transiently downregulated in 3D culture, whereas downregulation persists in 2D culture. Cluster 2 is transiently upregulated in 2D and 3D culture. Cluster 3 is transiently upregulated in 3D culture, whereas expression remains elevated in 2D culture throughout the culture period. See Table 2 for the associated pathway analysis. F) Mean‐centered sigma‐normalized heatmap of expression levels of key hepatocyte (metabolic enzymes and secreted factors) and progenitor cell markers. G) Stacked column plot showing the number of alternative splicing events during 3D spheroid cultures at day 1 and day 3 compared to mature PHH (FDR <0.05, ΔPSI >10%; see Experimental Section). Note that the extent of alternative splicing peaked at day 1 in agreement with the transcriptional upregulation of the splicing machinery (Table 1).

In ultra‐low attachment plates, PHH suspensions continuously aggregated over the course of 1 week to form compact spheroidal aggregates (**Figure** **1**A,B). To comprehensively profile the phenotypic changes on a molecular level we first used RNA‐Seq timecourse analysis. In total, 7419 genes were differentially expressed during spheroid formation after stringent multiple testing correction (FDR <0.01; Figure 1C), corresponding to 59% of all genes that were expressed in our data set. Surprisingly, when we compared transcriptomic alterations during spheroid formation to changes observed upon plating on collagen‐coated plastic (polystyrene), we found that the extent of transcriptomic changes in 2D and 3D culture were strikingly similar after 1 day of culture (5030 differentially expressed genes in 3D compared to 5153 differentially expressed genes in 2D; Figure 1D,E). However, the number of differentially expressed genes (DEGs) rapidly declined in spheroid culture, paralleling the reinforcement of cell–cell contacts with only 55 DEGs once compact spheroids were formed after 7 days. In contrast, the number of DEGs decreased considerably less in 2D monolayer culture with 3812 genes being differentially expressed at the same time point (Figure 1D).

Genes that were transiently downregulated during spheroid aggregation were enriched in metabolic enzymes (FDR <10^−15^) and complement and coagulation factors (FDR <10^−15^), and included important hepatic markers, such as cytochrome P450s (CYPs), alcohol dehydrogenases (ADHs) and UGT1A1, as well as important secretory proteins, such as albumin, ApoB, prothrombin (F2), and TTR (Figure 1F; **Table** **2**). In contrast, transiently upregulated genes were strongly enriched in the molecular machinery involved in ribosome (FDR = 2.4×10^−9^) and proteasome (FDR = 3.8×10^−8^) components, as well as Hippo signaling (FDR = 1×10^−4^). Furthermore, hepatocytes transiently increased the expression of genes involved in transcriptomic remodeling including RNA transport (FDR <10^−15^), transcription (FDR = 7.2×10^−6^), and splicing (FDR <10^−15^).

**Table 2:** Differentially regulated pathways in 2D monolayer and 3D spheroid culture of primary human hepatocytes. Only five differentially regulated pathways per cluster are shown. For the complete list of differentially regulated pathways we refer to Table S3, Supporting Information. FDR = false discovery rate

Cluster	Pathway	Number of genes	Enrichment ratio	*p*‐value	FDR
Cluster 1	Metabolic pathways	1021	2,12	<1×10^−15^	<1×10^−15^
	Complement and coagulation cascades	60	8,8	<1 × 10^−15^	<1 × 10^−15^
	Valine, leucine, and isoleucine degradation	43	7,12	2 × 10^−16^	2 × 10^−14^
	Fatty acid degradation	40	7,4	4 × 10^−16^	4 × 10^−14^
	Drug metabolism	59	4,58	9 × 10^−10^	3 × 10^−8^
Cluster 2	RNA transport	149	1,93	<1 × 10^−15^	<1 × 10^−15^
	Spliceosome	126	2,26	<1 × 10^−15^	<1 × 10^−15^
	Ribosome	129	1,88	6 × 10^−15^	6 × 10^−13^
	Proteasome	41	2,32	4 × 10^−11^	3 × 10^−9^
	Cell cycle	92	1,46	4 × 10^−2^	1 × 10^−2^
Cluster 3	Regulation of actin cytoskeleteon	133	2,48	3 × 10^−10^	9 × 10^−8^
	Endocytosis	199	1,94	3 × 10^−7^	3 × 10^−5^
	Focal adhesion	125	2,2	4 × 10^−7^	3 × 10^−5^
	Adherens junction	63	2,73	8 × 10^−7^	5 × 10^−5^
	Phosphatidylinositol signaling	70	2,36	2 × 10^−5^	1 × 10^−3^

To evaluate the functional consequence of these alterations we performed transcriptome‐wide analyses of alternative splicing. Most alternative splicing events occurred after day 1 in culture, coinciding with the peak of differential gene expression (Figure 1G; Table S4, Supporting Information). Alternative splicing was observed for the core components of the Hippo pathway, including YAP1, TAZ, NF2, MST1, and MST4, consistent with previous analyses of liver regeneration.^[^ ^19^ ^]^ Importantly, we observed a transient increase in markers for reprogramming and hepatic progenitor states, such as EPCAM, SOX9, KLF4, and KRT19 in 3D but not in 2D monolayer culture (Figure 1F).

Combined, these results indicate that isolation from the native in vivo microenvironment and loss of cell–cell contacts rather than plating constitutes the driving force behind the rapid phenotypic decline of PHH in culture. Furthermore, they demonstrate that the molecular phenotype of hepatocytes in 3D culture is highly dynamic and thus provide a unique paradigm to study human hepatocyte plasticity.

### 3.2 Transcriptomic and Proteomic Dynamics during Spheroid Aggregation Recapitulate the In Vivo Hepatocyte Regeneration Program

**Figure 2:**
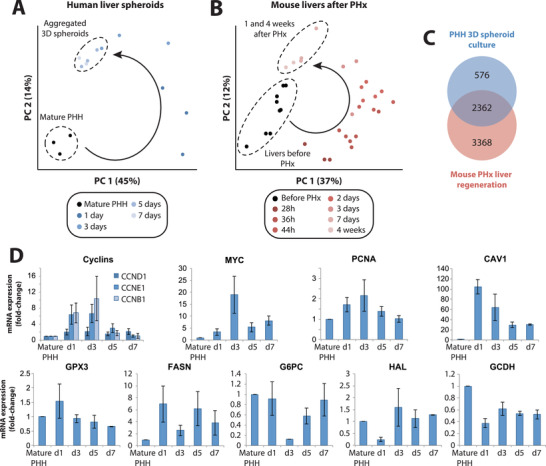
Transcriptomic dynamics during spheroid aggregation closely resemble the regenerative program after partial hepatectomy in mice in vivo. Principal component analyses of transcriptomic changes A) in human liver spheroids and B) in mouse liver regeneration after partial hepatectomy reveal strong similarities. Specifically, hepatocytes in both paradigms undergo substantial transient changes of transcriptomic signatures followed by redifferentiation to a mature phenotype. C) Venn diagram showing the overlap between the genes that are differentially expressed during PHH spheroid aggregation and mouse liver regeneration (F‐tests, *q* < 0.01; see Experimental Section). D) Expression patterns (FPKM fold‐changes compared to mature PHH) of proliferation markers (MYC and PCNA) and metabolic genes (CAV1, GPX3, FASN, G6PC, HAL, GCDH) during PHH spheroid aggregation. *N* = 3 different donors. Error bars indicate S.E.M.

Next, we investigated how the molecular changes during spheroid aggregation of human hepatocytes relate to liver regeneration in vivo. First we compared our transcriptomic data set to available time series data from murine PHx models.^[^ ^20^ ^]^ In both data sets we observed massive changes in expression patterns within the first 3 days, which were however only transient, as the transcriptomes after 7 days closely resembled those from mature human hepatocytes or mouse livers before PHx, respectively (**Figure** **2**A,B). Strikingly, 83% (3230 out of 3882) of human transcripts with mouse homologues that were differentially regulated during spheroid formation, showed similar alterations during mouse liver regeneration (Figure 2C). These genes were strongly enriched in RNA transport, spliceosome, and ribosome components, as well as cell cycle markers (FDR <0.01 for all; Table S5, Supporting Information). In contrast, analysis of genes exclusively regulated in the mouse after hepatectomy only identified the complement system as differentially regulated (FDR = 0.04), whereas no differentially regulated pathway was found for those genes exclusively regulated in human spheroids (FDR = 1 for all pathways). Combined, these results argue that the pathway activity dynamics identified during 3D spheroid formation recapitulate changes during liver regeneration in vivo.

Specifically, during aggregation PHH spheroids activated the cell cycle markers *CCND1*, *CCNE1*, and *CCNB1* (Figure 2D) and showed significant increases in expression of proliferation markers *MYC* and *PCNA*. PHH furthermore recapitulated alterations in expression patterns of metabolic mediators seen in liver regeneration, such as upregulation of *CAV1*, *GPX3*, and *FASN* as well as downregulation of *G6PC*, *HAL*, and *GCDH*, as previously shown in mice in vivo.^[^ ^21^, ^22^, ^23^ ^]^

**Figure 3:**
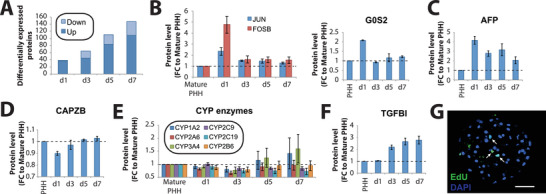
Primary human hepatocytes in 3D culture recapitulate key events of in vivo hepatocyte regeneration at the proteomic and cellular level. A) Bar plot showing the number of differentially expressed proteins (defined as *p* < 0.05 and fold‐change >2) compared to mature primary human hepatocytes at each time point. Expression pattern of proteins involved in B) acute response (JUN and FOSB; left panel), proliferation (G0S2; right panel), and C) dedifferentiation (AFP) peak transiently whereas D) the capping protein CAPZ and E) proteins involved in mature hepatic functions, including various CYP enzymes, show an inverse profile. F) Protein levels of TGF*β*I increased after 3 days and onward. Error bars indicate S.E.M. *N* = 3 different donors. G) EdU staining of hepatocyte spheroids after 7 days of culture shows few cells that underwent DNA replication. Scale bar = 100 µm.

To further our understanding of the parallels between spheroid formation and liver regeneration at the molecular level, we performed quantitative proteomics using tmt‐labeling. In total we identified 7794 proteins of which only 147 differed in abundance during spheroid aggregation compared to mature hepatocytes (*p* < 0.05; fold‐change >2; **Figure** **3**A, Figure S1, Supporting Information). The extent of proteomic changes is in agreement with previous proteomic analyses of mouse liver regeneration upon PHx^[^ ^24^, ^25^ ^]^ and the lower number of differentially regulated proteins compared to transcripts is likely at least in part due to longer protein half‐lives, which buffer changes in mRNA levels.

The first 24 h of 3D culture were characterized by acute injury signals, such as an increase in JUN and FOSB, as well as an upregulation of G0S2, a key regulator of cell cycle entry (Figure 3B). Furthermore, we observed a transient increase in the dedifferentiation markers as AFP, which during liver regeneration are specifically expressed in proliferating oval and transitional cells (Figure 3C).^[^ ^26^ ^]^ In agreement with these findings, the actin capping protein CAPZB, a regulator of hepatic Hippo signaling activation and maintenance of the differentiated hepatocyte state,^[^ ^27^ ^]^ was transiently downregulated (Figure 3D). These expression increases are paralleled by the downregulation of various metabolic enzymes characteristic of mature hepatocytes, including CYP1A2, CYP2A6, CYP2C9, CYP2C19, CYP2B6, and CYP3A4 (Figure 3E), in agreement with previous observations of transient downregulation of drug metabolizing enzymes and transporters at the transcript level.^[^ ^28^ ^]^ In addition, we saw an increase in TGF*β*I after 3 days (Figure 3F), in agreement with the peak of TGF*β* synthesis observed in vivo 3 days after PHx.^[^ ^29^ ^]^

Next, we asked whether these transcriptomic and proteomic dynamics translated into regenerative responses at the cellular level. To this aim, we evaluated proliferation using EdU labeling. We observed robust staining confirming active DNA synthesis in a fraction of cells (Figure 3G). In light of these data, we conclude that PHH recapitulate the key molecular and cellular events of the in vivo hepatocyte regenerative program within few days of culture.

### 3.3 Multidimensional Omics Analysis Identifies Critical Factors for Human Hepatocyte Regeneration

Even though PHH enter a regenerative program during 3D spheroid formation, hallmarked by dedifferentiation, changes in metabolic enzyme expression and cell cycle entry, the extent of proliferation remained considerably lower than in PHx mouse models. To explore the underlying causes, we performed a global analysis of transcription factor activity using our transcriptomic data to infer promoter motif activity (see Experimental Section).^[^ ^17^ ^]^

**Figure 4:**
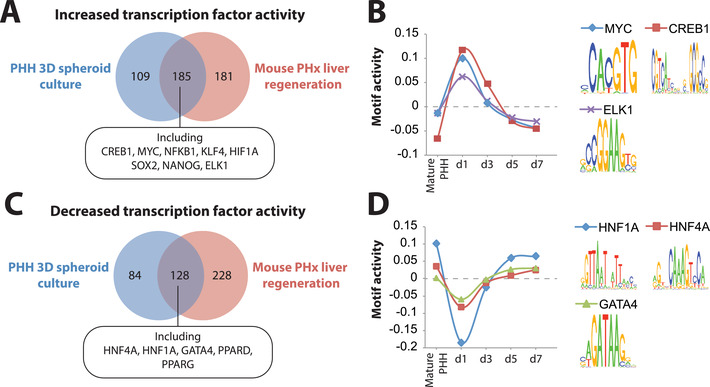
Global activity analysis of transcriptional regulatory motifs reveals high concordance between human hepatocyte spheroid culture and mouse hepatectomy models. Transcription factors (TFs) with A) transiently increased and C) transiently decreased activity signatures within the first 3 days largely overlap between human liver spheroids and in mouse partial hepatectomy models of in vivo liver regeneration. B) Examples of TFs whose activity transiently increases include MYC, CREB, and ELK1, whereas D) TFs controlling mature hepatic differentiation, such as HNF1A, HNF4A, and GATA4, are transiently downregulated. The corresponding binding motif sequence logos are shown for each TF in (B) and (D).

First, we validated the approach by focusing specifically on those TFs whose activity transiently increased or decreased during liver regeneration and spheroid aggregation, respectively (Table S6, Supporting Information). Importantly, these analyses revealed largely overlapping profiles. 63% (185 of 293) of TFs that were activated within the first 72 h during spheroid aggregation of human hepatocytes in vitro showed similar profiles during liver regeneration in the mouse, including multiple factors associated with early response to injury, such as HIF1A and JUN, stemness, such as MYC, SOX2, and NANOG, as well as Notch and growth factor signaling mediators, including HES1, ELK, CREB, and SP1 (**Figure** **4**A,B; Figure S2, Supporting Information). Similar overlap (60.3%) was observed for the pattern of TFs whose activity was transiently downregulated, including various factors involved in the maintenance of a differentiated phenotype, such as HNF1A, HNF4A, and GATA4 (Figure 4C,D).

**Figure 5:**
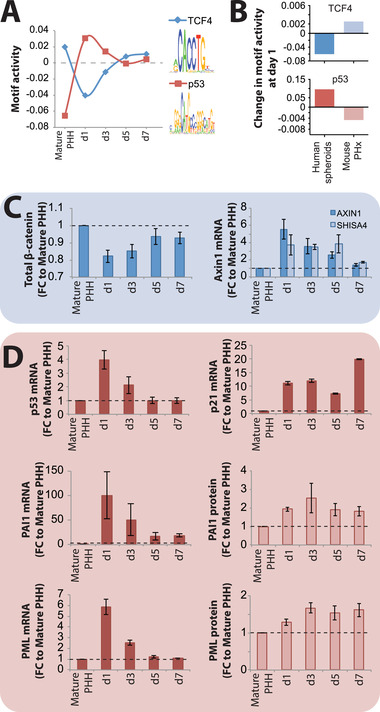
TCF4 and p53 activity are deregulated during spheroid aggregation. A) Transcription factor activity analysis revealed that activity of TCF4 (blue) transiently decreased during spheroid formation, indicative of inactive canonical Wnt signaling. In contrast, activity of p53 (red) increased during culture. B) Activity of the murine orthologues of TCF4 and p53 increases and decreases, respectively, during liver regeneration 1 day (28 h) after partial hepatectomy. C) During human spheroid aggregation, protein levels of the Wnt signaling mediator *β*‐catenin transiently decreased, whereas transcript levels of the negative Wnt signaling regulators AXIN1 and SHISA4 increased during spheroid aggregation, thus corroborating the observed lack of Wnt signaling activation. D) Transcript levels of p53 and its canonical targets p21, PAI1, and PML increase during spheroid aggregation, supporting the activation of p53 identified by motif activity profile analysis. *N* = 3 different donors. Values on the y‐axes indicate fold‐changes compared to mature PHH. Error bars indicate S.E.M.

Encouraged by these results, we then explored signaling axes that might explain the limited extent of proliferation observed in PHH spheroids. To this end, we focused on TFs whose activities differed between PHH and mice during early stages of regeneration. Strikingly, activity of TCF4, the main transcriptional effector of Wnt/*β*‐catenin signaling during liver regeneration,^[^ ^30^ ^]^ was among the TFs whose activity was strongly downregulated during spheroid aggregation (**Figure** **5**A,B). In contrast, activity of p53, which is tightly regulated in multiple tissue regeneration models^[^ ^31^, ^32^ ^]^ and abrogates hepatocyte proliferation in vivo,^[^ ^33^ ^]^ increased (Figure 5A,B).

To confirm these findings, we analyzed whether transcriptomic and proteomic patterns were corroborating TCF4 and p53 pathway activity data. During spheroid aggregation, protein levels of total *β*‐catenin decreased, expression of the negative Wnt‐regulators AXIN1 and SHISA4^[^ ^34^ ^]^ increased and the *bona fide* Wnt‐target genes AXIN2 and LGR5 remained undetected at both, protein and transcript level (Figure 5C). In contrast, expression of p53 and its target genes p21 (CDKN1A), PAI1 (SERPINE1),^[^ ^35^ ^]^ and PML^[^ ^36^ ^]^ increased on transcript and protein level (Figure 5D). We thus hypothesized that lack of Wnt/*β*‐catenin pathway activation and induction of the p53 signaling axis might underlie the limited hepatocyte proliferation during spheroid aggregation.

### 3.4 Activation of Wnt/*β*‐Catenin Signaling Inhibits p53 and is Sufficient to Drive Proliferation of Primary Human Hepatocytes in 3D Culture

**Figure 6:**
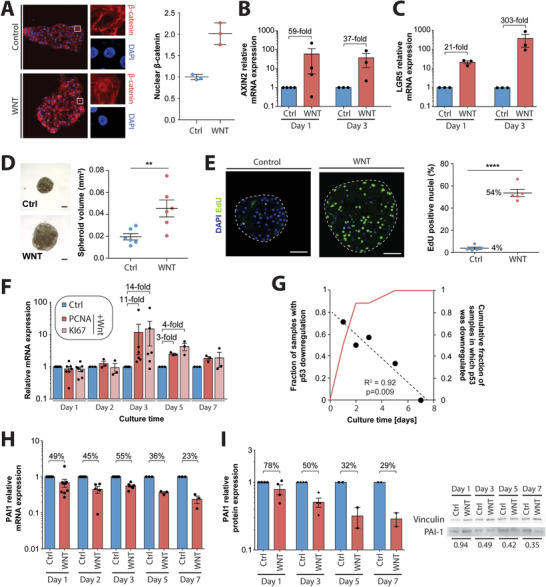
Activation of Wnt/*β*‐catenin signaling inhibits p53 and amplifies proliferation of primary human hepatocytes. A) Treatment of PHH spheroids with 3 µm of the GSK3*β* inhibitor Chir99021 (labeled as “WNT”) rapidly induces nuclear translocation and cytoplasmic accumulation of *β*‐catenin, as well as induction of the *bona fide* Wnt signaling target genes B) AXIN2 and C) LGR5. *N* = 3–4. D) Chir99021 treatment is sufficient to drastically increase spheroid volume after 7 days of culture (*p* < 0.01; heteroscedastic paired *t*‐test). Note that representative brightfield images are shown in scale. E) Induction of Wnt signaling results in substantially increased hepatocyte proliferation (*p* < 0.0001; heteroscedastic paired *t*‐test). Scale bar = 100 µm. F) Chir99021 treatment causes induction of the proliferation markers PCNA and Ki67. G) The increased proliferation is paralleled by downregulation of p53. Notably, the dynamics of p53 inhibition differ between donors. The fraction of samples in which p53 was downregulated (defined as > twofold) was highest after 1 day and downregulation clearly correlated with culture time (indicated by dots and dashed regression corresponding to the left ordinate; *R* ^2^ and *p*‐value based on Pearson correlation). However, while p53 was downregulated in every sample (*n* = 8), trough levels were reached as late as day 5 in one sample (indicated by red line corresponding to the right ordinate). Expression of the anti‐proliferative p53 target gene PAI1 at H) transcript and I) protein level. See Figure S3, Supporting Information for full Western blot images. Expression levels are shown relative to the untreated control at the same time point. *N* = 2–8 independent experiments using two to four different donors. ** and **** corresponds to *p* < 0.01 and *p* < 0.0001, respectively.

To functionally validate the importance of Wnt/*β*‐catenin signaling for hepatocyte regeneration, we treated cells with the GSK3*β* inhibitor Chir99021 (Wnt/*β*‐catenin signaling activator). Notably, Chir99021 treatment resulted in nuclear translocation and a twofold increase of unphosphorylated (i.e., active) *β*‐catenin, as well as robust activation of the canonical *bona fide* Wnt‐target genes AXIN2 and LGR5 (**Figure** **6**A–C). Importantly, spheroids treated with Chir99021 during the aggregation process were significantly larger in size (2.2‐fold) than untreated spheroids (Figure 6D). To scrutinize whether this increase in size was primarily driven by hepatocyte proliferation, we performed EdU incorporation assays and found that 53.6 ± 3% (SEM) of nuclei were labeled, compared to 3.9 ± 0.9% in untreated controls (*p* < 0.0001; Figure 6E). Furthermore, we observed clear induction of the proliferation markers Ki67 and PCNA, which peaked between 3 days (*n* = 3/6) and 5 days (*n* = 3/6) in culture (Figure 6F).

Importantly, activation of Wnt/*β*‐catenin signaling blunted the increase in p53 expression and repressed the induction of its anti‐proliferative target PAI1 on both transcript and protein level (*p* < 0.05; Figure 6G–I; Figure S3, Supporting Information). Notably, the dynamics of p53 inhibition differed between donors and 50% (*n* = 4/8), 37.5% (*n* = 3/8), and 12.5% (*n* = 1/8) reached through levels after 1, 2, and 5 days, respectively. While timing was inter‐individually different, p53 was downregulated > twofold in all donors tested (Figure 6G). Interestingly, the extent of p53 downregulation correlated well with the degree of PCNA induction in the same sample (Spearman *ρ* = 0.9; Figure S4, Supporting Information), corroborating the hypothesis of a mechanistic link between p53 repression and proliferation. Combined, these results indicate that activation of Wnt/*β*‐catenin signaling during spheroid formation inhibits the p53‐PAI1 signaling axis and is sufficient to induce proliferation of the vast majority of human hepatocytes.

## 4 Discussion

While the capacity of the human liver to regenerate has been known for millennia,^[^ ^37^ ^]^ insights into the cellular and molecular processes of liver regeneration stem from animal models or, more recently, human organoid cultures.^[^ ^6^, ^7^ ^]^ Here, we present an accessible 3D in vitro model of primary liver cells in which human hepatocytes recapitulate defining events of the in vivo regenerative program without the need for cell selection, scaffolding, or complex growth factor treatment regimens, thus providing an accessible paradigm to study the molecular biology of hepatocyte regeneration in a human cell system.

The early phases of the regenerative program were characterized by modulation of basic cellular functions. Ribosomal biogenesis was significantly increased after 24 h (Table 2), mirroring findings made by seminal studies of the regenerating rat liver.^[^ ^38^ ^]^ Similarly, the early phase was characterized by an upregulation of the transcriptional machinery, indicative of hypertranscription, which is associated with proliferating progenitor cells and is a well‐known phenomenon in other experimental paradigms of vertebrate organ regeneration, such as regrowth of salamander limbs following amputation.^[^ ^39^ ^]^ We furthermore observed significant differences in alternative splicing that overall affected 1278 genes (Figure 1G). Specifically, we found that the isoform composition of YAP1 and other Hippo signaling mediators was significantly altered, which facilitates regeneration in mouse livers.^[^ ^19^ ^]^ In addition, we detected significant remodeling of the actin cytoskeleton and transient repression of the actin capping protein CAPZB, suggesting differences in actomyosin contractility and mechanotransduction, which have been shown to enable hepatocyte dedifferentiation and proliferation in vivo.^[^ ^27^ ^]^

Integrative multi‐layered analysis of transcriptomic, proteomic and TF activity data revealed that while growth factor, Hippo and Notch‐signaling were activated during the spheroid formation process, activation of Wnt/*β*‐catenin signaling was lacking. Importantly, these findings were consistent with murine data showing that Kupffer cells are the major source of Wnt ligand secretion and mice lacking the ability to secrete Wnts from macrophages displayed cell cycle defects and decreased TCF4 activation during liver regeneration following PHx.^[^ ^40^ ^]^ Thus, one can speculate that culture paradigms lacking non‐parenchymal cells require extrinsic activation of Wnt/*β*‐catenin signaling to initiate cell cycle entry.

In vivo, Wnt/*β*‐catenin signaling cooperates with growth factor signaling^[^ ^41^ ^]^ and promotes hepatocyte proliferation in homeostasis,^[^ ^42^ ^]^ as well as during liver regeneration in a variety of models.^[^ ^43^ ^]^ Interestingly, Chir99021 has not been shown to be sufficient to drive proliferation of human hepatocytes in 2D culture.^[^ ^44^ ^]^ Notably, lack of functional hepatic Wnt signaling only delays liver regeneration but does not abrogate it. Our data recapitulate these observations for the first time in a human context. Without Wnt stimulation <10% of hepatocytes proliferated after 7 days (Figure 6D), closely resembling the reduced proliferation after PHx in hepatic *β*‐catenin^[^ ^45^ ^]^ or LGR4/5 knock‐out mice.^[^ ^46^ ^]^ In contrast, when Wnt/*β*‐catenin signaling was activated, >50% of PHH entered the cell cycle, which is similar to the extent of proliferation observed in vivo.^[^ ^47^ ^]^

Interestingly, our data suggest that the pro‐proliferative effect of Wnt/*β*‐catenin signaling is mediated, at least partially, by repression of p53. Increased hepatic p53 levels in a knock‐out mouse model of Mdm2, an E3 ubiquitin ligase that degrades p53, results in drastically reduced hepatocyte regeneration upon PHx,^[^ ^33^ ^]^ whereas hepatic p53 knock‐out results in increased hepatocyte proliferation rates in a mouse model of acetaminophen‐induced liver injury.^[^ ^48^ ^]^ Furthermore, in cancer cells p53 has been reported to repress *β*‐catenin,^[^ ^49^ ^]^ as well as its transcriptional co‐activator TCF4.^[^ ^50^ ^]^ While a direct link between Wnt/*β*‐catenin and p53 signaling during liver regeneration has to our knowledge not yet been reported, in cancer cell lines, Wnt/*β*‐catenin signaling increases expression of miR‐552, which targets p53 and promotes proliferation.^[^ ^51^ ^]^ Of note, regulation of p53 signaling is orchestrated by a plethora (>300) of post‐translational modifications that, together with context‐dependent cues, control target gene activation.^[^ ^52^, ^53^ ^]^ Elucidation of the precise molecular events underlying the regulatory cross‐talk between Wnt/*β*‐catenin and p53 signaling in liver regeneration thus remain an interesting area for future research.

In conclusion, we present a novel experimental 3D model of primary adult human liver cells to study the molecular and cellular biology of human hepatocyte regeneration. Using time series multi‐omics profiling in this paradigm we identified canonical Wnt signaling as a critical inhibitor of p53 that is sufficient to drive proliferation of the majority of human hepatocytes *ex vivo*. As such, these findings shed light on the mechanisms underlying human hepatocyte plasticity and can provide a versatile and scalable complement to organoids for hepatocyte expansion.

## Conflict of Interest

V.M.L. is founder, CEO, and shareholder of HepaPredict AB. In addition, V.M.L. discloses consultancy work for EnginZyme AB.

## Supporting information

Supporting Information

Supplemental Video 1

Supplemental Video 2

Supplemental Video 3

Supplemental Video 4
