# Supplementary material for: A 3D Cell Culture Model Identifies Wnt/β‐Catenin Mediated Inhibition of p53 as a Critical Step during Human Hepatocyte Regeneration
Source: Adv Sci (Weinh). 2020 Jun 23;7(15):2000248. doi: 10.1002/advs.202000248 (PMC7404138; doi:10.1002/advs.202000248)
Supplement: Supplementary file 1 — Supporting Information [file ADVS-7-2000248-s001.pdf]

© 2020 Wiley-VCH GmbH

ADVANCED SCIENCE
Open Access

Supporting Information

for *Adv. Sci.*, DOI: 10.1002/advs.202000248

A 3D culture model identifies Wnt/β-catenin mediated inhibition of p53 as critical for human hepatocyte regeneration

Nuria Oliva-Vilarnau, Sabine U. Vorrink, Magnus Ingelman-Sundberg and Volker M. Lauschke

A 3D culture model identifies Wnt/β-catenin mediated inhibition of p53 as critical for human hepatocyte regeneration

Nuria Oliva-Vilarnau, Sabine U. Vorrink, Magnus Ingelman-Sundberg and Volker M. Lauschke

Corresponding author (*):

Volker Lauschke, Department of Physiology and Pharmacology, Karolinska Institutet, SE-171 77 Stockholm; mail: volker.lauschke@ki.se; Tel +46852487711

Table of contents

Fig.S1..2

Fig.S2..4

Fig.S3..5

Fig.S4..6

Table S1..7

Table S2..8

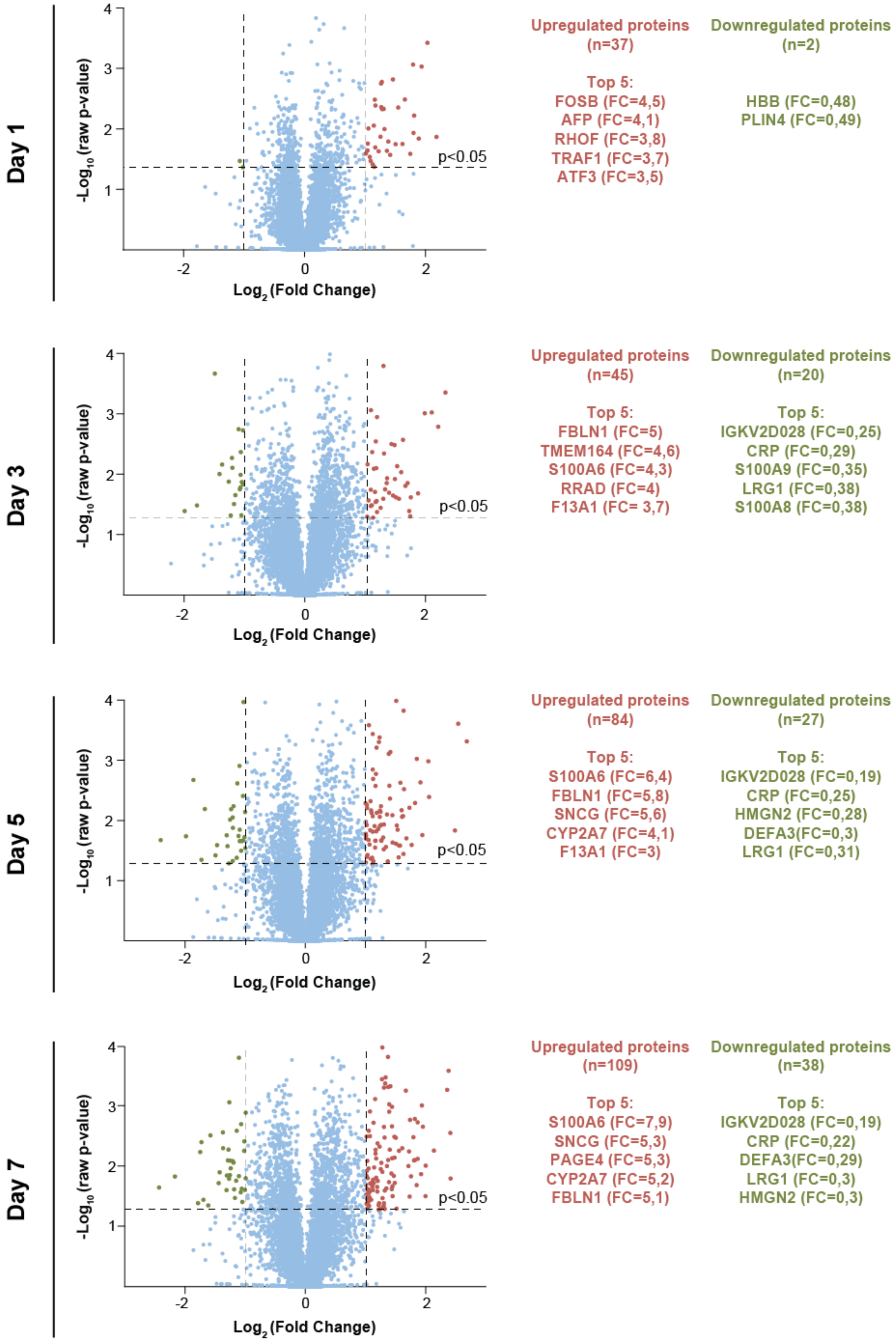
Day 1
-Log10 (raw p-value)
Log2 (Fold Change)
p<0.05
Upregulated proteins
(n=37)
Top 5:
FOSB (FC=4,5)
AFP (FC=4,1)
RHOF (FC=3,8)
TRAF1 (FC=3,7)
ATF3 (FC=3,5)
Downregulated proteins
(n=2)
HBB (FC=0,48)
PLIN4 (FC=0,49)
Day 3
-Log10 (raw p-value)
Log2 (Fold Change)
p<0.05
Upregulated proteins
(n=45)
Top 5:
FBLN1 (FC=5)
TMEM164 (FC=4,6)
S100A6 (FC=4,3)
RRAD (FC=4)
F13A1 (FC= 3,7)
Downregulated proteins
(n=20)
Top 5:
IGKV2D028 (FC=0,25)
CRP (FC=0,29)
S100A9 (FC=0,35)
LRG1 (FC=0,38)
S100A8 (FC=0,38)
Day 5
-Log10 (raw p-value)
Log2 (Fold Change)
p<0.05
Upregulated proteins
(n=84)
Top 5:
S100A6 (FC=6,4)
FBLN1 (FC=5,8)
SNCG (FC=5,6)
CYP2A7 (FC=4,1)
F13A1 (FC=3)
Downregulated proteins
(n=27)
Top 5:
IGKV2D028 (FC=0,19)
CRP (FC=0,25)
HMGN2 (FC=0,28)
DEFA3(FC=0,3)
LRG1 (FC=0,31)
Day 7
-Log10 (raw p-value)
Log2 (Fold Change)
p<0.05
Upregulated proteins
(n=109)
Top 5:
S100A6 (FC=7,9)
SNCG (FC=5,3)
PAGE4 (FC=5,3)
CYP2A7 (FC=5,2)
FBLN1 (FC=5,1)
Downregulated proteins
(n=38)
Top 5:
IGKV2D028 (FC=0,19)
CRP (FC=0,22)
DEFA3(FC=0,29)
LRG1 (FC=0,3)
HMGN2 (FC=0,3)

Supplementary Figure 1: Proteomic time series analyses of 3D spheroid cultures of primary human hepatocytes. Volcano plots and the top 5 most differentially expressed proteins are shown at every time point (n=3 donors per time point).

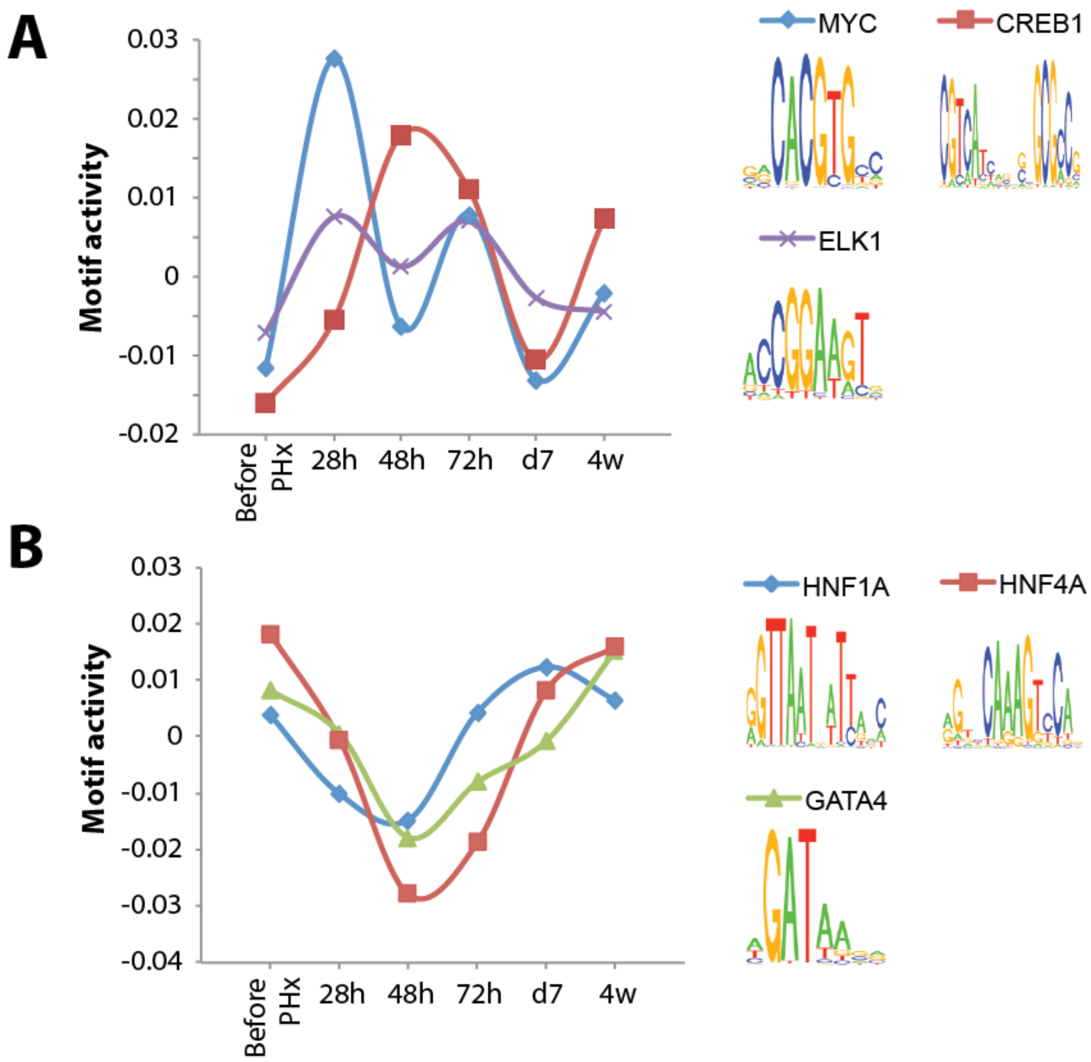

Supplementary Figure 2. Activity profiles of key transcription factors in mice during regeneration following partial hepatectomy. Activities of MYC, CREB and ELK1 transiently increase (**A**), whereas transcription factors controlling mature hepatic phenotypes, such as HNF1A, HNF4A and GATA4, are transiently downregulated (**B**). These profiles align well with their counterparts during human 3D spheroid formation (compare Figure 4).

Supplementary Figure 3

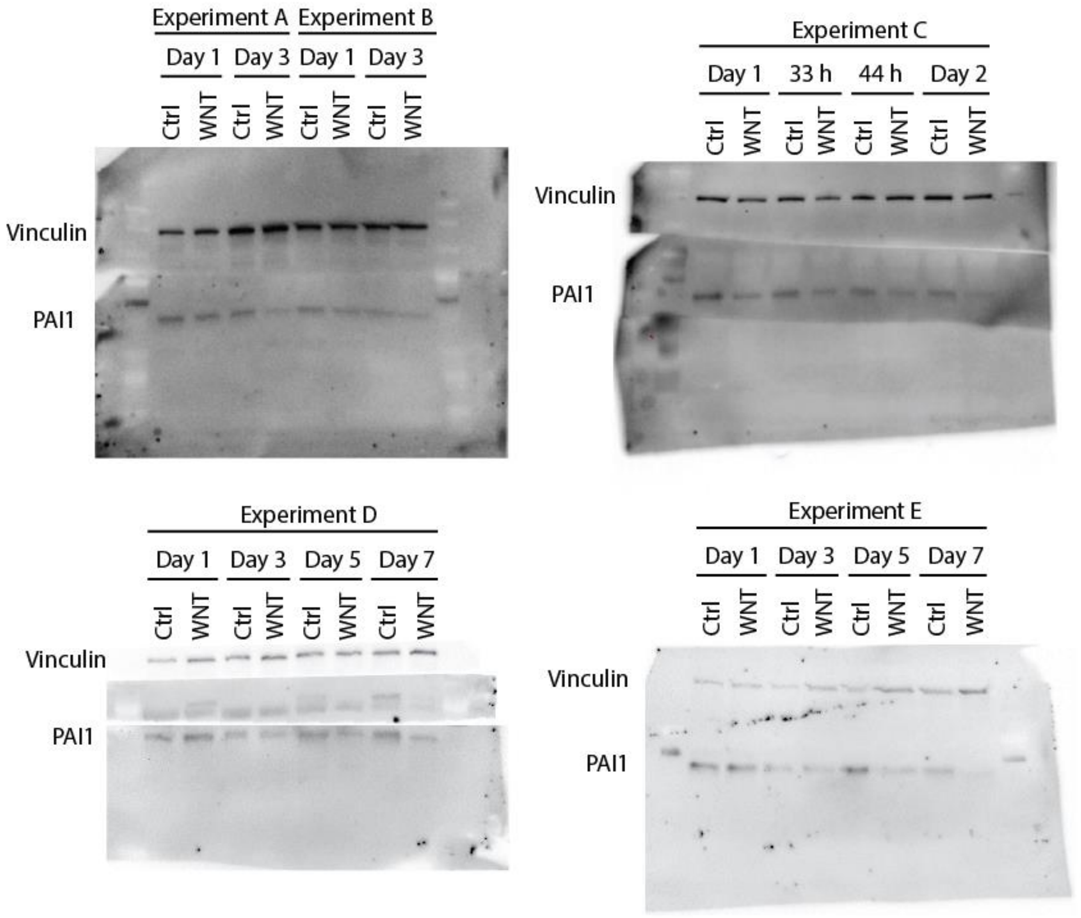

Supplementary Figure 3. Full Western blots showing reduction in PAI1 protein levels upon GSK3β inhibition (Wnt/β-catenin signaling activation).

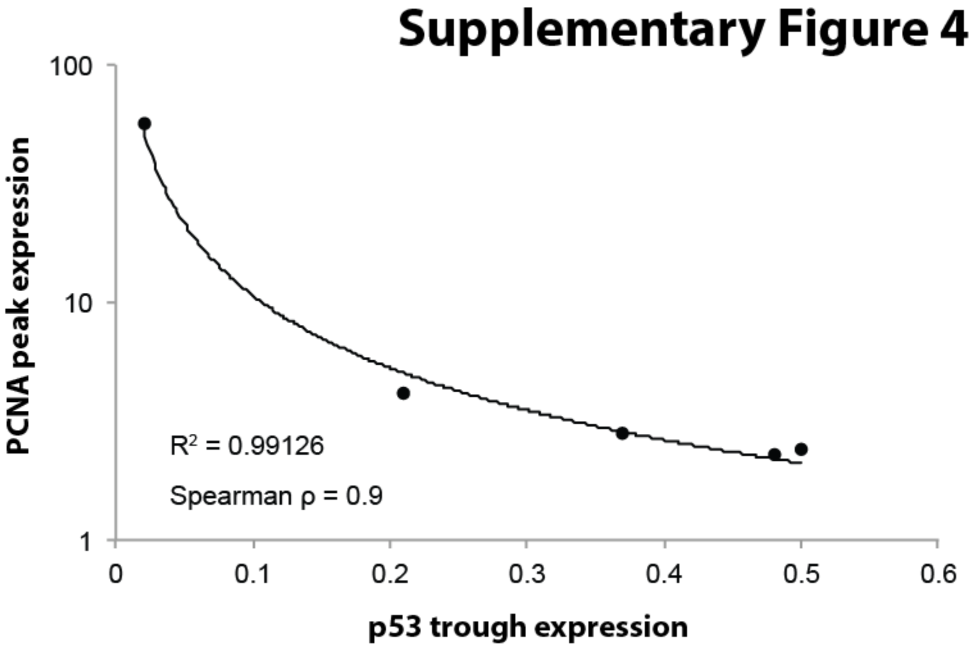

Supplementary Figure 4. p53 trough expression is a good predictor of PCNA peak levels.

Supplementary Table 1: Taqman assay probes for qPCR analyses.

Gene probed	Probe ID
TBP	Hs00427620_m1
PCNA	Hs00427214_g1
MKI67	Hs04260396_g1
TP53	Hs01034249_m1
PAI1(SERPINE1)	Hs00167155_m1

Supplementary Table 2: Specifications and working dilutions of antibodies for Western blots and immunofluorescence. IF = immunofluorescence; WB = Western blot.

Target gene	Host species	Working dilution (method)	Manufacturer	Catalog #
B-catenin	Mouse	1:500 (IF)	BD Biosciences	610153
PAI1 (SERPINE1)	Rabbit	1:500 (WB)	Abcam	Ab66705
Vinculin	Rabbit	1:10000 (WB)	Abcam	ab129002
Anti-rabbit-HRP	Goat	1:5000 (WB)	DAKO	P0448
